# Thoracic Fracture Adjacent to Spinal Fusion Following Cardiopulmonary Resuscitation: A Case Report

**DOI:** 10.7759/cureus.86656

**Published:** 2025-06-24

**Authors:** Mia Liebeskind, Jamie E Clarke, Jimmy C Huang

**Affiliations:** 1 Department of Radiology, University of California Los Angeles, Los Angeles, USA

**Keywords:** ct, hyperextension vertebral fracture, instrumented spine fusion, mri, msk radiology, neuro radiology, radiograph, spinal mri, thoracic spine fracture, traumatic cpr

## Abstract

Cardiopulmonary resuscitation (CPR), an important life-saving procedure implemented in dire situations, carries a risk of traumatic injuries. One such injury that may arise from the chest compressions performed during CPR is a bone fracture. Although the most commonly involved fracture locations are the ribs and sternum, spinal fractures may also occur. In this case report, the authors share a unique case of a distraction-hyperextension thoracic spine fracture in a patient who had an inferiorly adjacent multilevel spinal fusion and subsequently endured a T9 fracture with anterior vertebral body distraction and intra-vertebral body hematoma formation after CPR. Through this case report, the authors aim to (i) describe the imaging findings of this thoracic spine injury and (ii) convey the pertinent medical history and mechanical factors of CPR that may have contributed to this fracture pattern. Increased awareness of potential spinal injuries from CPR and understanding their mechanism will aid in detection and timely management when evaluating a patient with back pain following CPR.

## Introduction

The chest compressions of cardiopulmonary resuscitation (CPR) often result in rib and sternal fractures and may be seen following CPR in approximately 50% and 25% of patients, respectively [1]. Spinal fractures are an uncommon but significant complication that can be increasingly detected with the rising utilization of CT/MRI. Bony demineralization in an aging population and decreased spinal mobility in patients with spondylosis, ankylosis, or surgical fusion further increase the risk of spinal trauma after CPR. In this case report, the authors aim to convey a clear medical history and a relevant clinical story for how a thoracic vertebral body fracture may occur during CPR, with multimodality imaging examples. In this case, we describe a rare CPR complication of a T9 distraction-hyperextension fracture in a 79-year-old man with a T10-L2 posterior-approach spinal fusion.

## Case presentation

**Figure 1:**
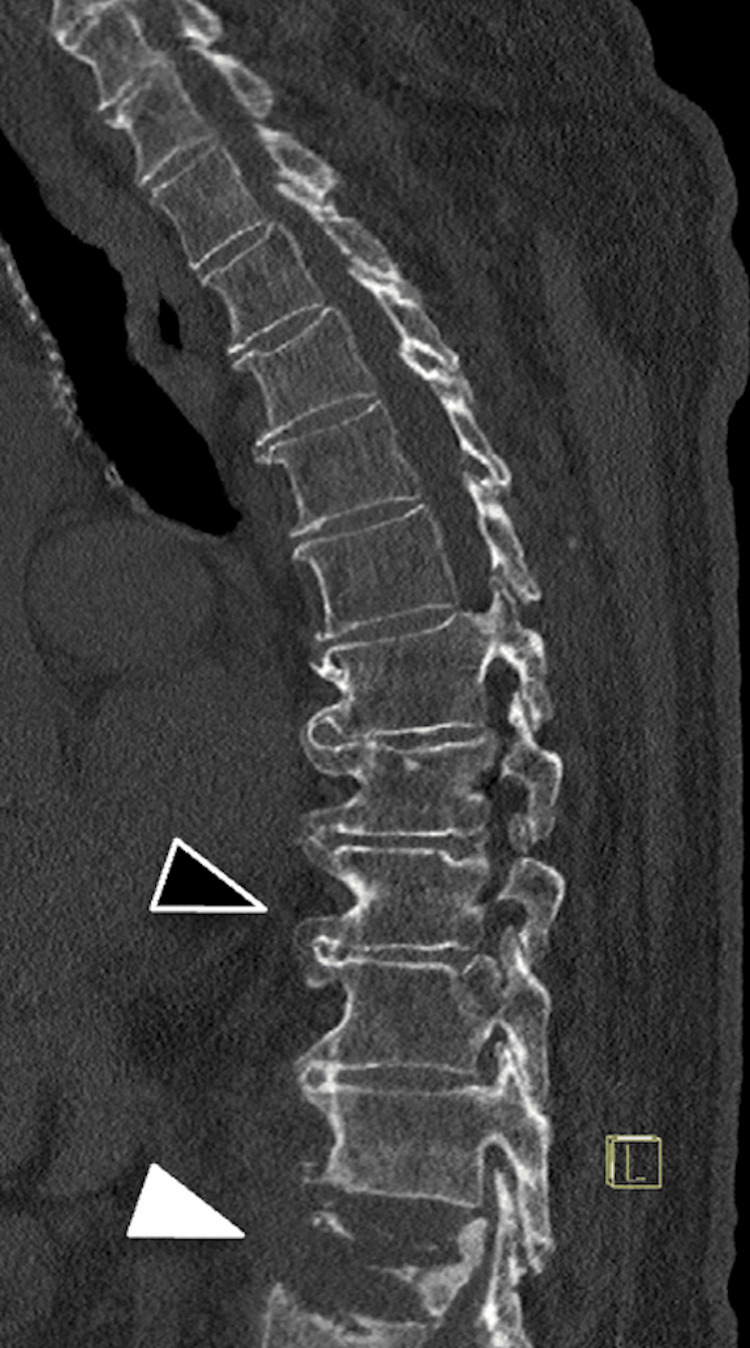
Preoperative CT thoracic spine. Preoperative CT thoracic spine of this 79-year-old man, acquired two days preoperatively, demonstrating a T12 compression fracture (white arrowhead), diffuse idiopathic skeletal hyperostosis (DISH) of the T7-T11 vertebrae, and a T9 vertebral body (black arrowhead) with no evidence of fracture or injury.

**Figure 2:**
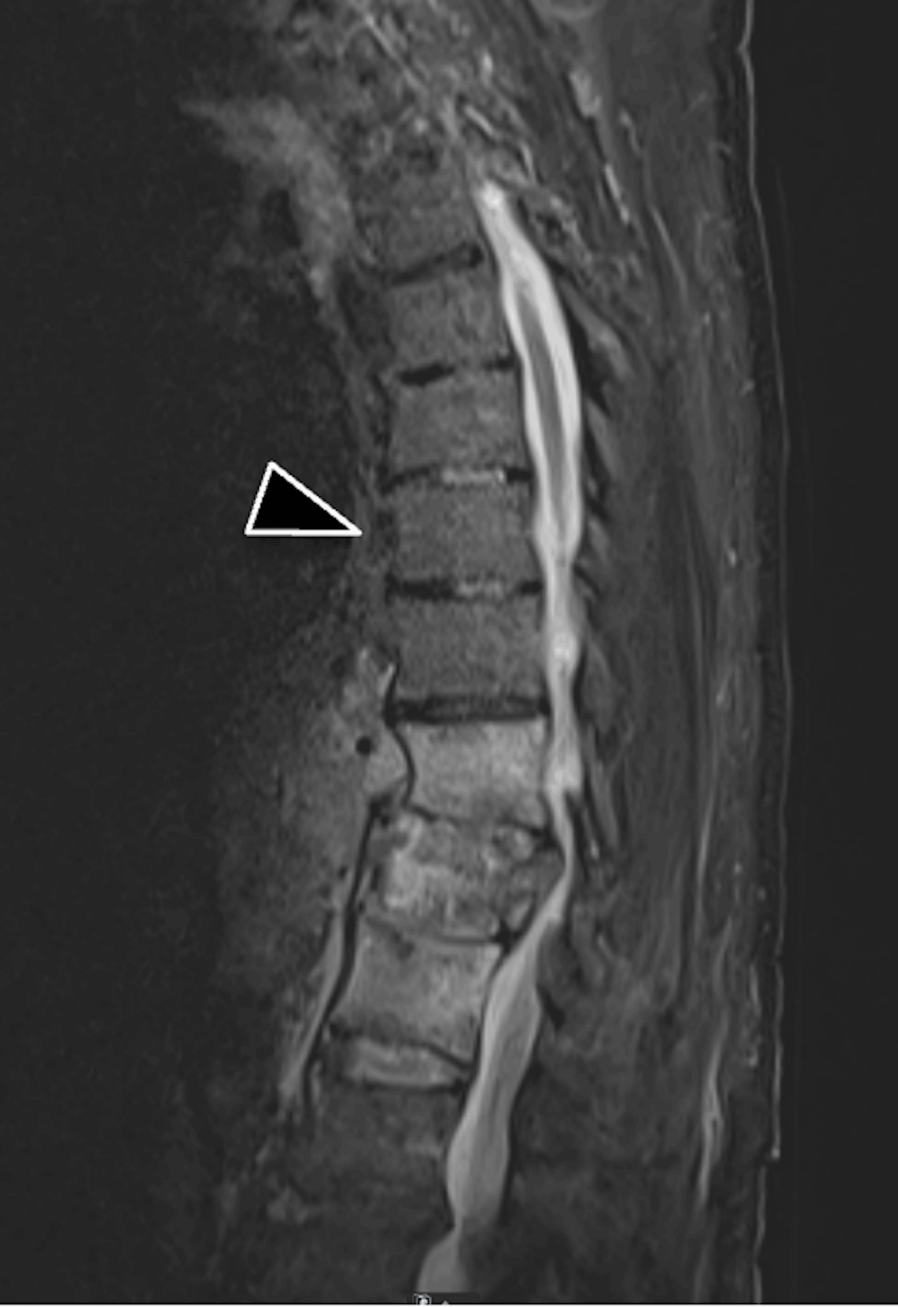
Preoperative T2W/STIR MRI thoracic spine. Preoperative T2-weighted (T2W)/Short Tau Inversion Recovery (STIR) MRI thoracic spine of this 79-year-old man, acquired two days preoperatively, demonstrating a T9 vertebral body (black arrowhead) with no evidence of fracture or injury.

A 79-year-old man with an extensive medical history, including heart failure, hypertension, hyperlipidemia, non-obstructive coronary artery disease, paroxysmal atrial fibrillation, obstructive sleep apnea, prior prostate cancer with a chronic suprapubic catheter, and a pathologic T12 compression fracture concerned to be pathologic presented to the hospital for an elective thoracolumbar spinal fusion to treat his chronic mid-back pain and to obtain a tissue sample of the T12 vertebral body for pathology evaluation of potential malignancy. The patient attended neurosurgery clinic, had a preoperative radiologic workup of his thoracic spine with computed tomography (CT; Figure 1) and magnetic resonance imaging (MRI; Figure 2), had a comprehensive preoperative workup to assess for surgical candidacy, and was deemed a candidate to proceed with an elective posterior spinal fusion from T10-L2.

The day before his scheduled surgery, the patient was admitted to the hospital to be optimized for the operation by the internal medicine team. He received stress-dose steroids, was consulted on by the endocrinology team, and received two units of red blood cells. On the day of his thoracolumbar spinal fusion surgery, the medical team felt he was optimized for surgery. He was appropriately consented regarding risks of the procedure and elected to proceed. He was taken to the operating room where he was given prophylactic intravenous (IV) Ancef, had sequential compression devices (SCDs) placed for deep vein thrombosis prophylaxis, and was anesthetized with general anesthesia. The neurosurgical team proceeded to perform a T11-T12 laminectomy, a T10-L2 posterior fusion, and a biopsy of the T12 vertebral body lesion. The neurosurgical procedure note commented that a few small complications occurred during surgery, including a small pleural breach at T11 that was repaired intraoperatively and a small durotomy with a cerebrospinal fluid (CSF) leak that was repaired intraoperatively. Findings noted during surgery included a T12 compression fracture, T12 retropulsion, and malignant-appearing tissue in the sampled T12 vertebral body.

After surgery, he remained mechanically ventilated and was moved from the operating room to the surgical intensive care unit (SICU). Given the violation and repair of his pleura during surgery, the thoracic surgeons consulted on him immediately postoperatively and obtained a chest X-ray (CXR), which revealed a tiny left apical pneumothorax but was otherwise unremarkable. Overnight following the day of his surgery, he had more difficulty breathing with increased respiratory requirements on the ventilator. A repeat CXR again showed the pneumothorax with interval development of left pleural effusion and atelectasis.

The following morning, the patient suffered a code and required CPR. The medical resident who responded to the code described that they were called to the patient’s bedside two minutes prior to the initiation of the code for reported desaturation of the patient to a blood oxygenation level in the low 80s despite the patient being on mechanical ventilation. At the bedside, the medical resident assessed the patient and observed an acute rapid drop in his oxygen saturation that progressed to the 70s. At that time, the patient was switched from mechanical ventilation to manual ventilation. Despite the ventilatory interventions, the patient continued to desaturate and then had a stark drop in blood pressure, with measured mean arterial pressures (MAPs) decreasing from the 60s range to the 20s range in the span of one minute. Pulses were not palpable at that time and upon a second check of the pulses for confirmation, the medical resident called a pulseless electrical activity (PEA) arrest code. The medical team in the SICU initiated chest compressions and placed electrical shock pads on the patient’s chest. Shortly after, the patient was administered 1 g of epinephrine and subsequently achieved return of spontaneous circulation (ROSC). The patient was then started on peripherally administered IV Levophed to elevate his blood pressure, had a right internal jugular line placed, and received a massive blood transfusion (given the concern of the clinical team for a left hemothorax). The medical team then placed a left-sided chest tube, which returned 200 mL of blood. At that time, it was deemed that the patient was stabilized for advanced imaging and further evaluation. The patient then had a CT scan of his chest, abdomen, and pelvis, which identified a large right pneumothorax. A second chest tube was subsequently placed in his right chest by the thoracic surgery team. When the CT results returned, the patient was found to have a new T9 fracture, with distraction of the anterior mid-vertebral body and an oblique fracture line extending from the anterior vertebral body with blood-density material filling the cavity of the fracture defect.

**Figure 3:**
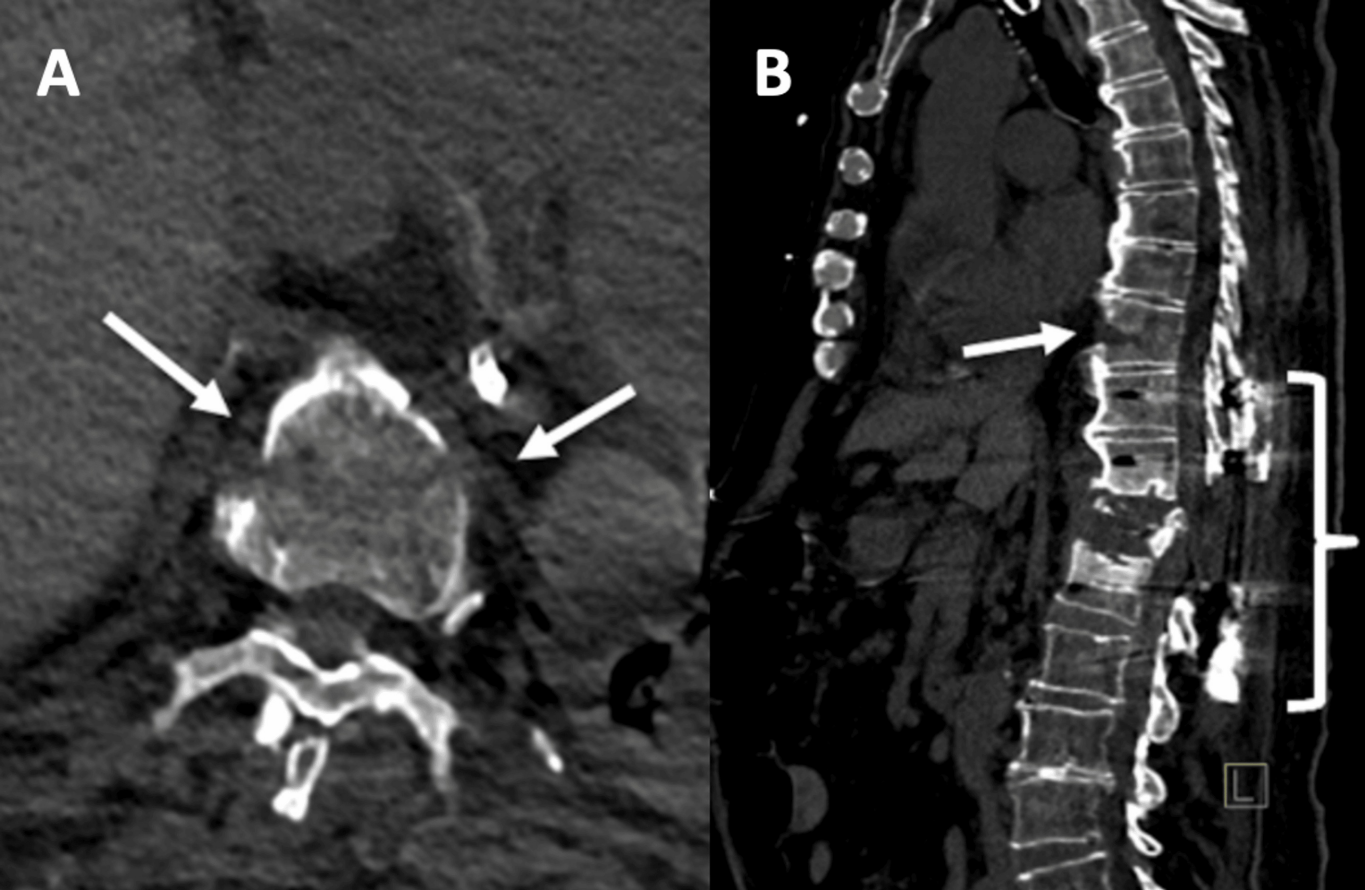
Postoperative CT thoracic spine in the bone window. Postoperative CT thoracic spine in the bone window with axial (A) and sagittal (B) planes for this 79-year-old man, acquired two weeks postoperatively, demonstrating a T9 vertebral body anterior cortex fracture (white arrows) with extension to the inferior endplate, hemorrhage along the fracture line with soft tissue density within the cavity of the fracture space, and T10-L2 fusion hardware (white brackets) surrounding the T12 pathologic compression deformity.

**Figure 4:**
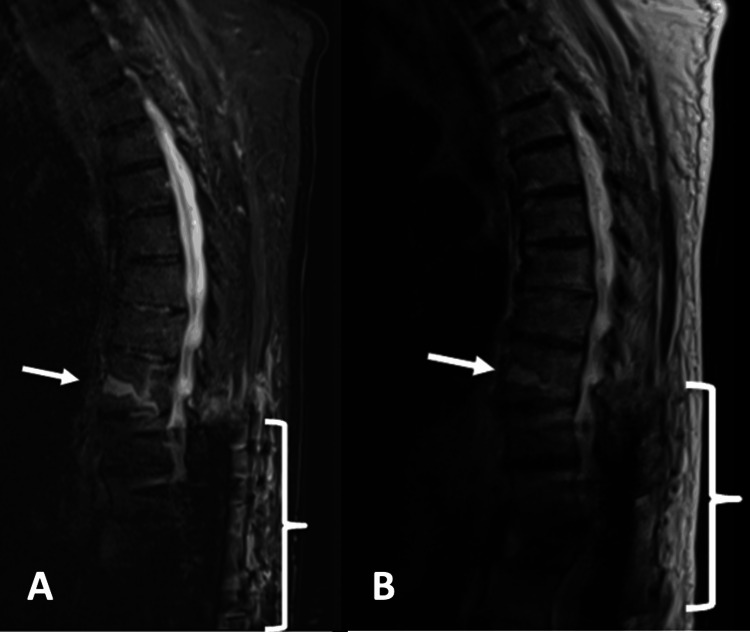
Postoperative T2W/STIR and T2W and MRI thoracic spine. Postoperative T2-weighted (T2W)/Short Tau Inversion Recovery (STIR) (A) and T2W (B) MRI thoracic spine of this 79-year-old man, acquired two weeks postoperatively, demonstrating a T9 vertebral body fracture with internal fluid material (white arrows), presumably hemorrhage, and T10-L2 fusion hardware (white bracket) surrounding the T12 pathologic compression deformity.

As the patient continued to recover from surgery, he began to slowly attempt more movement. He first attempted sitting up in bed on postoperative day (POD) 9 and first worked to get out of bed with the support of a physical therapist on POD 12. Upon attempting to get out of bed and ambulate, the patient endorsed excruciating mid-back pain, which he stated was still midline but slightly higher up along the back than his preoperative pain. The neurosurgical team obtained a dedicated thoracic spine CT (Figures 3A, 3B) and MRI (Figures 4A, 4B) scans at the two-week postoperative mark to better assess his post-surgical healing and the T9 vertebral body injury. The dedicated thoracic imaging more clearly demonstrated the T9 vertebral body injury. The MRI in particular provided a more detailed characterization of hemorrhagic material within the vertebral body fracture line.

**Figure 5:**
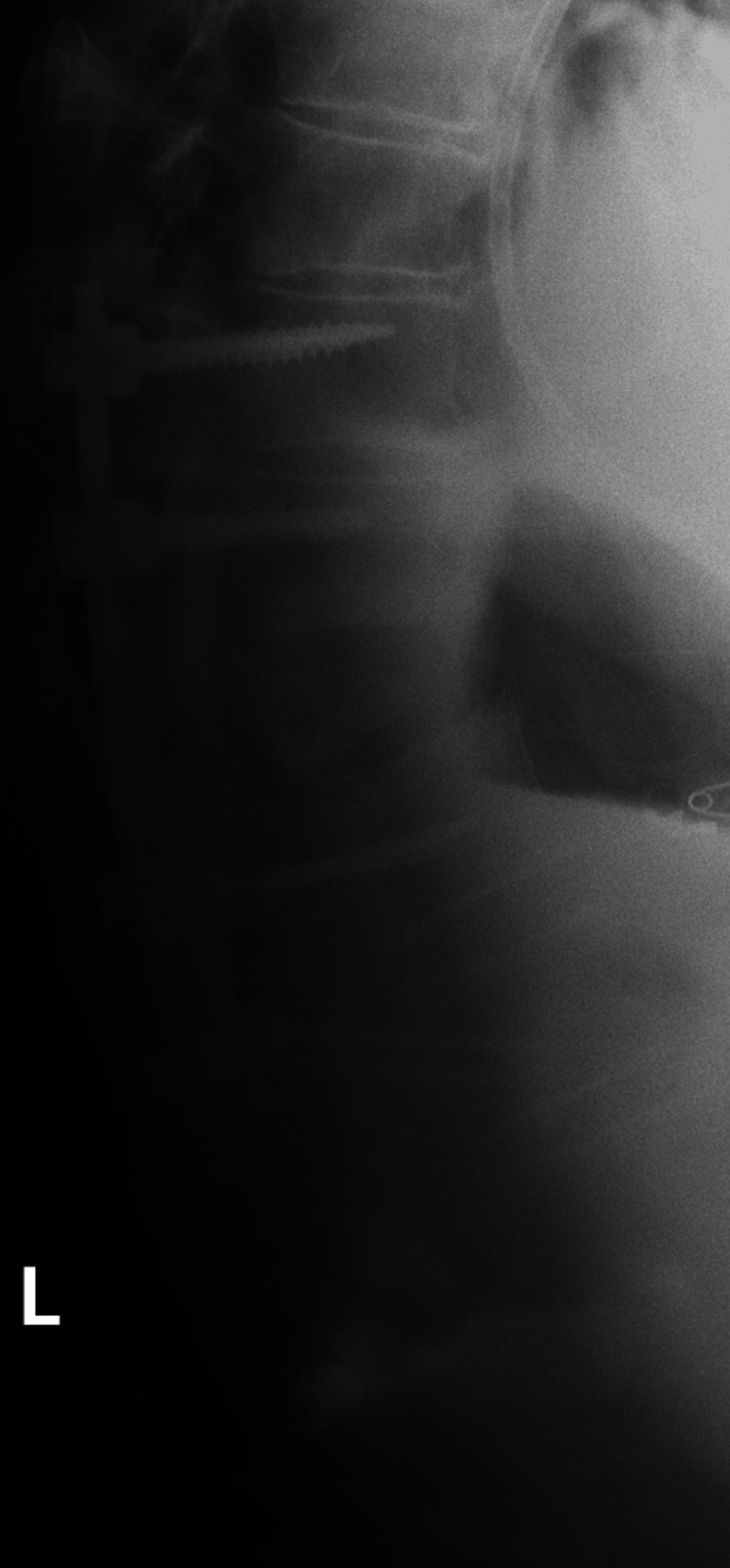
Radiograph of the thoracic spine. Radiograph of the thoracic spine of this 79-year-old man, acquired four weeks postoperatively, demonstrating lucency in the anterior mid-vertebral body of T9, consistent with the patient’s known T9 vertebral body reverse Chance fracture, and T10-L2 fusion hardware surrounding the T12 pathologic compression deformity.

Following identification of this entity by the radiologist, the neurosurgical team created precautionary protocols for the patient’s physical therapy, ordered short interval radiographic follow-up (Figure 5), and developed a plan for monitoring the patient to be prepared if there were to become a need for repeat surgical intervention.

## Discussion

This unique case of a distraction-hyperextension T9 fracture after CPR includes close-interval CT and MRI imaging immediately before and after the injury and presence of hemorrhagic blood product within the fracture line. These attributes helped confirm that the timing of injury was during CPR. In addition, this patient’s T10-L2 fusion may have increased vulnerability of the thoracolumbar junction that combined with CPR chest compressions to result in a T9-level injury.

Quality chest compressions are an integral part of effective CPR but are commonly associated with bony trauma. Manual chest compressions vary biomechanically depending on many uncontrolled factors, for example, the side of approach or force/pressure applied by the rescuer. The downward force applied on the thorax during chest compressions hyperextends the thoracic spine and increases motion at the thoracolumbar junction, increasing risk of injury at this level. Numerous case reports have described thoracic spine injury after manual [2-5] and mechanical [5-7] compressions. Review of the case reports in the literature indicates that these injuries occur mostly in the lower thoracic spine and thoracolumbar junction. The CT and/or MRI findings of transdiscal anterior thoracic spine distraction or distraction of anterior vertebral body fragments in these cases correlate with the distraction-hyperextension mechanism described above [3,4,6,8].

Thoracolumbar injuries are classified by the thoracolumbar injury classification and severity score (TLICS) which includes fracture appearance and clinical features to create an encompassing numerical score that is used to determine treatment. The AO spine classification system for thoracolumbar injuries is based on imaging features of fracture morphology and neurological status without treatment determination. Within the AO spine classification, the B type morphology encompasses distraction injuries, and the B3 injury, the most severe, is the distraction-hyperextension pattern that tears the anterior longitudinal ligament with extension of the force either through the disc or vertebral body or the disc, resulting in a widened intervertebral height or anterior distracted fracture line through the vertebral body. This fracture pattern could be called a “reverse Chance fracture” in contrast to the hyperflexion Chance fracture which has a horizontal fracture line that distracts the posterior vertebral body and elements.

Thoracic hyperextension injuries are known to occur with increased frequency in patients with DISH and ankylosing spondylitis due to the increased rigidity of the fused spine [8,9]. Similarly, decreased spinal mobility after surgical fusion results in rigidity and longer lever arm of the fused level. The result is altered biomechanics at adjacent segment levels that increase range of motion and stress, generally worsening with fusion length, that correlate with the frequent clinical observation of increased degeneration, instability and injury at adjacent levels [10].

## Conclusions

In summary, CPR is commonly performed in the medical setting yet carries the risk of bony trauma, specifically, spinal injury during CPR usually occurs at the thoracolumbar junction from a distraction-hyperextension mechanism. Patient factors that create spinal rigidity and alter spinal biokinetic forces, especially at the thoracolumbar junction, increase the risk of vertebral body or transdiscal spinal trauma. This case highlights that spinal fusion, known to alter biomechanics at the adjacent spinal levels, may heighten spinal susceptibility during CPR, and awareness of this clinical picture can aid physicians and caregivers in the timely recognition and management of this rare but important complication.
